# Design and Experimental Approach to the Construction of a Human Signal-Molecule-Profiling Database

**DOI:** 10.3390/ijerph10126887

**Published:** 2013-12-09

**Authors:** Xinyan Zhao, Tao Dong

**Affiliations:** Department of Micro and Nano Systems Technology (IMST), Faculty of Technology and Maritime Sciences (TekMar), Vestfold University College (HiVE), Tønsberg, N3103, Norway; E-Mail: td@hive.no

**Keywords:** public health informatics, data acquisition, health data management, signal molecule profiling, lab on a chip

## Abstract

The human signal-molecule-profiling database (HSMPD) is designed as a prospective medical database for translational bioinformatics (TBI). To explore the feasibility of low-cost database construction, we studied the roadmap of HSMPD. A HSMPD-oriented tool, called “signal-molecule-profiling (SMP) chip” was developed for data acquisition, which can be employed in the routine blood tests in hospitals; the results will be stored in the HSMPD system automatically. HSMPD system can provide data services for the TBI community, which generates a stable income to support the data acquisition. The small-scale experimental test was performed in the hospital to verify SMP chips and the demo HSMPD software. One hundred and eighty nine complete SMP records were collected, and the demo HSMPD system was also evaluated in the survey study on patients and doctors. The function of SMP chip was verified, whereas the demo HSMPD software needed to be improved. The survey study showed that patients would only accept free tests of SMP chips when they originally needed blood examinations. The study indicated that the construction of HSMPD relies on the self-motivated cooperation of the TBI community and the traditional healthcare system. The proposed roadmap potentially provides an executable solution to build the HSMPD without high costs.

## 1. Introduction

Biological information stored in databases is important to understand the complex relations between genes, proteins, environmental factors and diseases. Research on biobanks and medical databases provide opportunities for a broad spectrum of interests and applications in translational bioinformatics (TBI). Many countries plan to invest in health IT research by constructing biobanks and health information databases [1]. These projects make rapid improvements in this field. The ethical, legal and social needs of studies are integral concerns of the entire scientific community. A significant bioinformatics infrastructure effort that aims to construct high-capacity public health information databases is underway in Europe and the United States. For example, Biobank Norway will spend more than 13 million USD on the establishment of a biobank-based research infrastructure [2]. Many similar joint efforts on biobank infrastructure and research are available, in which governments around the world fund activities. Population-based health surveys and research biobanks gain much attention from TBI researchers and substantial international funding, such as the National Institute of Health in the United States, which is the largest funder of basic biomedical research in the world. Investments in the public health system enable large-scale surveys on medical issues to collect data and biological materials from large populations. The results of these surveys with the corresponding biomedical data or samples often turn into international open resources for TBI research. The Janus Serum Bank is an example of a useful biobank [3]. In this biobank, which includes 170,000 clinical samples, blood samples reserved for cancer research were collected through Norwegian health screening studies. Researchers in the field of biology, medical science, bioinformatics and health care systems will benefit from those types of biomedical databases and biobanks. For example, Thomas *et al.*, applied bioinformatics approaches to identify pathways related to human leukemogens by using the Kyoto Encyclopedia of Genes and Genomes database [4].

Health information technology (HIT), one of the pillars in future medical systems, is based on high capacity and qualified biomedical databases [5]. Nowadays, physical examination results are analysed only by individual doctors. Future HIT will provide additional references of auxiliary diagnoses by contrasting these diagnoses with past clinical cases in the database. The IT service will substantially increase the efficiency and reliability of the medical diagnosis without increasing costs. Personalized Medicine and Telehealth may be achieved along with the development of HIT. The development of HIT usually begins with the construction of large biomedical databases [6,7,8]. Of course, some small-scale medical databases were also helpful to the public health professionals [9].

Various biomedical databases have already been constructed worldwide, such as Human Genome Database (HGD), Recon 2, DrugBank, Human Metabolome Database, and ClinicalTrials [10,11,12,13,14,15]. Most of these databases are open to researchers free of charge. The prominent development of bioinformatics depends on the availability of biomedical databases. However, the existing databases mainly focus on laboratory studies instead of clinical studies. The completion and maintenance of these databases mainly depend on research funds from the government. Therefore, the current biomedical databases are still far from clinical auxiliary diagnoses. Few clinical databases have been established, such as ClinicalTrials, which currently lists 144,133 studies with locations in 50 states and 185 countries [15]. However, a large part of those studies achieved poor results. Reliable clinical databases are still in highly demand in TBI.

These biomedical databases can be roughly classified into three categories, according to the origin and properties of data. The first category, represented by ClinicalTrials [15] and Virus Pathogen Database and Analysis Resource (ViPR) [16], which data came from biological studies or clinical trials is accumulated by scientific experiments. The scientific experiments were always designed for specific purposes, and the test objects were always selected. Consequently, the experimental data were restricted to a specific target field and had been investigated thoroughly before being included in the database. Not surprisingly, these multifarious data have little residual value for TBI researchers. The second category of biomedical database comes from gigantic scientific projects, such as the Human Genome Project (HGP) [13]. These prospective databases were directly designed for fundamental research, instead of a small-scale scientific topic. Before the data of genes were sequenced on a large-scale and collected, the standard data structure and file formats were defined in advance. Besides, the data acquisition in HGP was separated from in-depth analysis of the data, so the database could expand at a breathtaking speed. The final database is standard and ready for data processing, which greatly improved the research methodologies of genetics and correlative disciplines [17]. However, the investment of HGP reached an unparalleled peak in the field of biology and medical science. The third category of biomedical database is accumulated by hospitals. Incomputable clinical data are generated in the global healthcare system every day. These valuable data are derived from real clinical cases, rather than from simulation tests on animal models. In fact, they were used data of doctors, which means the costs of data acquisition have been majorly covered by the healthcare system. When the clinical data are translated to electronic health records (EHRs) in a public database, TBI studies will be greatly stimulated [18]. Unfortunately, these clinical databases often raise concerns about the privacy of patients, and are subject to multiple protections by laws and ethics rules. Moreover, the medical records are often irregular due to the multifarious treatment processes of different patients. Many researchers have conducted a large amount of pilot studies in clinical knowledge management. However, most of these studies have not entered into clinical use because of the high cost of data acquisition and the traditional work habits of doctors and nurses [19,20].

**Figure 1:**
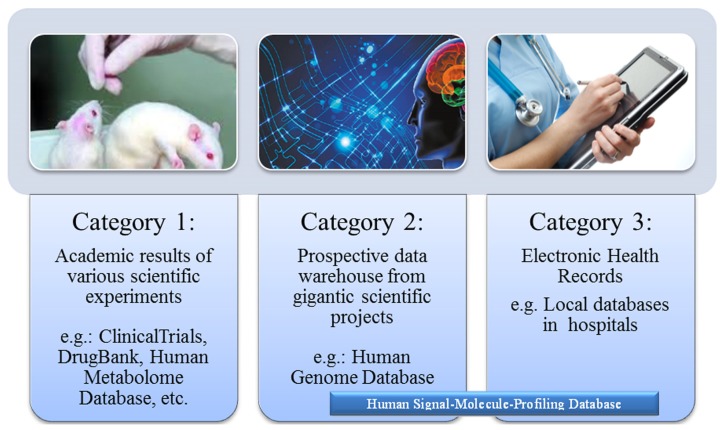
Three categories of current biomedical databases. The current biomedical databases can be roughly classified into three categories according to the origin and properties of data. HSMPD was proposed to take advantage of the healthcare system to build a prospective database for TBI. HSMPD contains large quantity of redundant information to doctors, but the information is valuable to TBI researchers.

HIT innovations are not urgent needs of doctors and nurses, and are not essential for patients either. Bioinformatics researchers seem to be the exclusive beneficiaries of EHRs at this stage. Therefore, two main strategies were proposed because of these circumstances. The first strategy is a compromise between the present healthcare systems and HIT, *i.e.*, traditional medical records will be converted into EHRs without changing the equipment and working habits of the existing system. The primal challenge of this strategy is the cost and efficiency of EHR conversion. The second strategy aims to redesign medical tools to generate automatically standard EHRs during the manipulation of doctors and nurses. It will change the machines, instead of the doctors and nurses, to avoid the conversion of medical records. According to the second strategy, the project of human signal-molecule-profiling database (HSMPD) was proposed to take advantage of the current healthcare system to build a standard prospective database as HGD. HSMPD is different from the first category of biomedical databases, because it will not be limited by any scientific experiment and will not select the donors of clinical data. HSMPD is also different from the third category of bio-databases, or a data warehouse of common EHR. The data in HSMPD are designed and standardized on the purpose of TBI, which contains large quantity of redundant information to doctors, (shown in Figure 1). Besides, the HSMPD project is required to solve both the ethical and economic problems at the same time.

In this study, a 1,536-chamber microfluidic chip prototype called “signal molecular profiling (SMP) chip” was developed as a specific tool for the data acquisition of HSMPD. It can standardize the detection of multiple signal molecules in one blood sample. Signal molecules, e.g., hormones and cytokines, are biochemical molecules released in one part of the body and deliver messages to affected cells in other parts. Although the concentrations of signal molecules are usually extremely low, signal molecules are important to maintain and regulate the functions of the human body. SMP can be regarded a composite order set of the human body that can provide holographic data of the entire human body. Moreover, SMP is related not only to human gene mapping, but also to the interactions between the human body and the environment [21]. The concentrations of signal molecules could reveal the status of personal health [22]. When SMP changes irregularly, some drastic activities have been or will be occurring in the body. In most cases, this dramatic change indicates the advent of disease (e.g., cardiovascular disease, cancer and invasion of pathogens) [23]. Much effort has been made to discover the diagnostic relationships between serological biomarkers and their respective diseases. However, the progress is hampered by the absence of a high-capacity clinical database on human serological information. Only a small proportion of such diagnostic knowledge has been uncovered, whereas most mapping relationships remain unknown [24]. The network of signal molecules has a well-ordered structure, which has been studied worldwide for decades [4,6,11]. Biomedical researchers divide signal molecules into several overlapping functional clusters. Nowadays, clusters of signal molecules have been clinically employed as diagnostic bases. Translational relationships of signal molecules are more complex than what is known in physiology or pathology. For example, understanding that lung diseases have incomprehensible effects on sex hormones is difficult [25]. Medical and biological researchers find it difficult to reveal thorough relationships inside the complex system, whereas TBI researchers are competent for this work if a qualified database can be established.

The SMP test can be regarded as an extended blood examination that aims to measure hundreds of cytokines and hormones by using a high-throughput tool. Given that the results of SMP tests can cover the information of regular blood tests in the hospitals, the SMP test can be accepted by doctors theoretically. Simultaneously, SMP records are good resources for multivariate analysis in TBI because SMP records embody large amounts of clinical information. Compared with other EHR systems, HSMPD is both an excellent gold mine for TBI and a compatible tool for traditional diagnosis systems.

Mass data stored in the medical system are considered the foundation of TBI. However, many preliminary attempts of TBI were unsuccessful because of misinformation. High-capacity databases with standardized health information are required. In fact, research on TBI has achieved considerable development on gene-related bioinformatics because of HGD. However, TBI, at the proteome level, is lagging behind because of the unavailability of corresponding databases. The HSMPD could be a good TBI resource; however, the high cost of data acquisition is a problem. In this study, we attempted to develop another possible solution to construct a low-cost HSMPD, by which the maturity of HSMPD is accompanied by the spontaneity in economics.

The SMP chip was developed for the construction of the HSMPD. This pilot study was a simulated validation in a real hospital to test the procedure of data acquisition in the HSMPD project. On the basis of the present conditions, we conducted a small-scale practical investigation on the data acquisition, storage and retrieval of SMP records in the demo HSMPD system. The feasibility and methodology of creating HSMPD was investigated and evaluated in a common hospital.

## 2. Experimental Section

### 2.1. The Roadmap for the HSMPD Project

**Figure 2:**
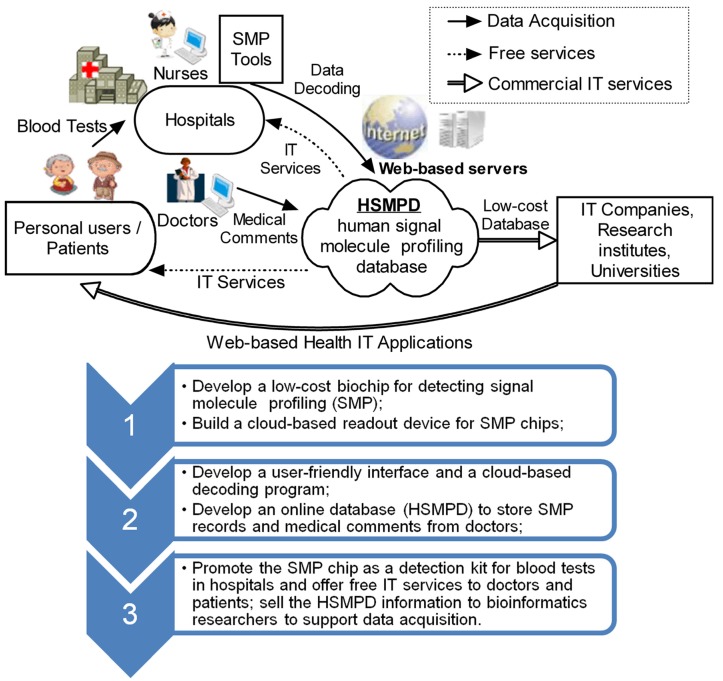
Technology roadmap of HSMPD construction. HSMPD is a bridge to connect traditional health care systems with the TBI community. The HSMPD services on TBI community can provide a stable income to support the data acquisition process of HSMPD, which forms a self-motivated mechanism.

To accelerate the development of public health systems, a low-cost solution is proposed to install the HSMPD without involving an enormous scientific project such as HGP. The key of the solution lies in the cooperation of IT and instrumentation engineering and the support of traditional health care systems and the TBI community (Figure 2).

The cost of data acquisition can be partially supported by the medical system because patients are supposed to pay for their original physical examinations. However, SMP chips are more expensive than traditional diagnostic reagents under the current circumstances. The deficit should be covered by public funding or be shifted to the database users in the TBI community. In this way, the high cost of data acquisition in HSMPD could be lowered to an acceptable amount for TBI researchers.

The initial step in the HSMPD project is to develop a HSMPD-oriented tool that should be standardized and high throughput to quantify hundreds of cytokines and hormones. To reduce the difficulty of data collection, SMP chips will be coupled automatically with the IT system. Furthermore, this tool should be as cheap as possible to be widely accepted by medical systems. When patients go to hospitals or health centres, SMP chips could perform the routine blood tests for hormones or cytokines because of the high efficiency and competitive price of the chips. SMP information will be decoded through a web-based program before relevant analysis reports are sent back. The SMP data will be stored in the web-based HSMPD at the same time. TBI researchers can obtain commercial information of the HSMPD at an acceptable cost, thus generating a stable income to cover the deficit during data acquisition. Once the HSMPD system is thoroughly developed, the serological criterion of SMP might become a popular multipurpose diagnostic tool. Chronic disease management, even the diagnosis of some mental diseases such as anxiety or depression, could benefit from SMP [26]. Without doubt, derivative HIT services from HSMPD will further encourage the growth of the TBI community.

### 2.2. The Design of Data Structure in the HSMPD

**Figure 3:**
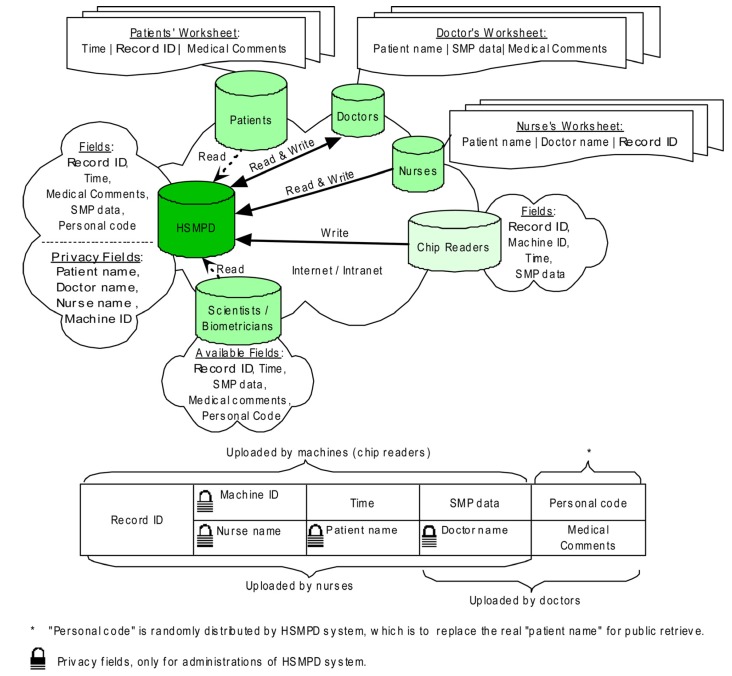
Data structure of HSMPD. Clinical records in HSMPD will be inputted by doctors, nurses and chip readers. The information of every record is categorized into two parts. The medical SMP data is open for all researchers and patients without unnecessary ethical review, whereas the private information of patients is protected behind firewall, which can be mediated by a trusted intermediary.

HSMPD adopts the framework of a relational database. Each SMP data set is divided into three interrelated sections with different information sources (Figure 3). The following data structure has been optimized for three times according to the comments of potential users. Figure 3 is the prioritization scheme at present.

The first section includes four fields: Record ID (RID), Patient’s Name (PN), Doctor’s Name (DN) and Nurse’s Name (NN). When patients go to the blood-collection room, nurses will check the information of the patients and input the patient records into the HSMPD system. Nurses are required to submit a one-time identity registration and login to the system before opening the Nurses worksheet. The NN field can be registered automatically when the worksheet is opened. However, the PN, DN and RID fields need to be filled-in manually. The RID is acquired from the serial numbers printed on the chip, which is the same as the exclusive record number of the corresponding SMP data.

The second section includes an SMP table and the following fields: RID, Machine ID (MID) and “Time”. This section will be filled by chip readers and related programs. However, we manually completed the entries in this study because the related machines were still under development. The SMP chip was designed to be readable in microplate readers, which are widely used in biomedical laboratories. The nurses took the blood samples of patients, and we operated SMP chips to load serum samples and diagnostic reagents. The SMP chip was read in a microplate reader to generate primary data. These manual operations of SMP chips could be automatically performed by future chip readers, which can also automatically read and record the RID of the chip and the time that the test was taken. The primary SMP data were decoded, and then stored in a SMP table. RID will also be registered in the SMP table. The chip reader submits the information to a web server, and the machine ID of the chip reader is reported. The exclusive RID will be the primary key that matches the information of the first section.

The third section, which will be mainly entered by doctors, includes three fields, namely, DN, medical comments (MC), and Personal Code of Patient (PCP). Doctors are also required to register before logging in. The DN field will be filled automatically when doctors open their worksheets. Besides, doctors are responsible for the private information of their patients. However, this information is not temporarily stored in HSMPD. The HSMPD system will generate PCP, the special code according to the private information of individual patients. The PCP, which is meaningless to doctors, is essential to the HSMPD because this code is the only information that TBI researchers can use to distinguish records of individual patients in the HSMPD. The merged data set from the first and second sections are presented in the corresponding doctor’s worksheet according to DN. Doctors need to check and recognize the patient information input by nurses. Three sections of that record will be related. The doctors need to add some medical comments in the SMP data of the patients. The MC section in the doctor’s worksheet is divided into two parts: the first part includes the symptom and complaints of the patient, which should be completed before conducting blood tests; the second part includes the diagnosis opinions. Both parts will be consolidated into the MC field, thus forming a complete SMP record.

The initial plan was to enable patients to register and login to the HSMPD system, in which the patients can visit their own SMP record. However, some doctors had a different opinion. The doctors were worried that their patients will experience unnecessary psychological stress if the patients misunderstood the SMP information. Therefore, the IT service for patients was not tested in this trial and was left for further studies.

This first system of HSMPD was realized by using Microsoft Access^®^ 2010 and shared on the intranet of the cooperating hospital to collect comments from invited doctors and nurses. Given that the utilized HSMPD is a general demonstration database, the structure and function of the demo system were limited. Based on the comments of users, the updated version of HSMPD system was developed by using Microsoft^®^ SQL Server^®^ 2005 while the user interfaces were written in C#(C Sharp) on Microsoft^®^ NET Framework^®^ 2.0.

### 2.3. Development of the SMP Chips

**Figure 4:**
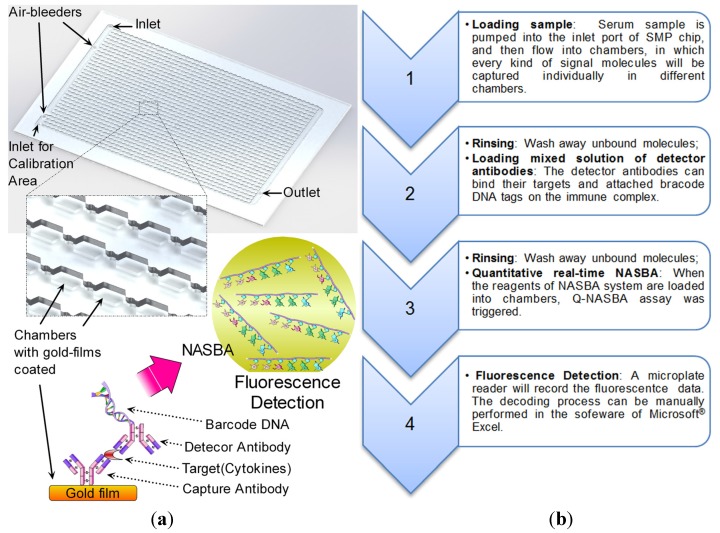
Structure of a SMP chip and its operation flow. (**a**) Gold films coated the surfaces of the poly (methyl methacrylate). Different capture antibodies for respective targets were immobilized on the gold film in every chamber. The principle of immuno-NASBA assay is similar to sandwich enzyme-linked immunosorbent assay (ELISA), however, the report group in the detector antibody is a barcode DNA with a T7 RNA polymerase promoter instead of an enzyme. The barcode DNA plays as a bridge to connect immunoassay and NASBA amplification. Then fluorescent signals will be generated in the NASBA amplification with the help of beacon probes. (**b**) The operation flow is proposed on the left.

The 1,536-chamber immuno-nucleic acid sequence-based amplification (immuno-NASBA) chip, named the “SMP chip”, was specifically designed for HSMPD data acquisition [26]. The SMP chip can be employed to simultaneously measure at most 1,344 kinds of hormones and cytokines in one serum sample (Figure 4). In fact, several versions of SMP chip prototypes were designed on the basis of previous research experience [27,28,29,30] and fabricated by using different materials and processes. The SMP chips here were fabricated in laminated poly(methyl methacrylate) slides by our laser engraving experience as described before [31,32].

In the SMP chip, 12.5% of total chambers are preserved for calibration tests. Therefore, only 1,344 chambers were left for monitoring targeted molecules. The sensing capability of the chip depends on an isothermal amplification assay, which is regarded as a combination of sandwich immunoassay and quantitative NASBA assay [33]. The sensitivity of the immuno-NASBA assay provides a convenient solution to quantify traces of signal molecules in samples. Similar to the previous immuno-NASBA chips, the SMP chip has the same dimensions as the 1,536-well microtiter plate, thus making the SMP chip readable in a microplate reader. The capability of the SMP chip was not completely exploited at the pilot stage. To demonstrate the SMP chip, 16 types of different capture antibodies were integrated on the chip to measure 16 types of signal molecule targets, including thyroid-stimulating hormone (TSH), tumor necrosis factor-alpha (TNF-α), cortisol, follicle-stimulating hormone (FSH), luteinizing hormone (LH), human chorionic gonadotropin (HCG), erythropoietin (EPO), gonadotropin-releasing hormone (GnRH), interferon-gamma (IFNγ), insulin-like growth factor 1 (IGF-1), epidermal growth factor (EGF), vascular endothelial growth factor (VEGF), interleukin (IL)-1 beta, IL-2, IL-6 and IL-8. Given that a specific chip reader was developing, the SMP chips were operated manually and read in a microplate reader as described in our previously publications [31,33].

### 2.4. Data Acquisition and Survey Study

**Figure 5:**
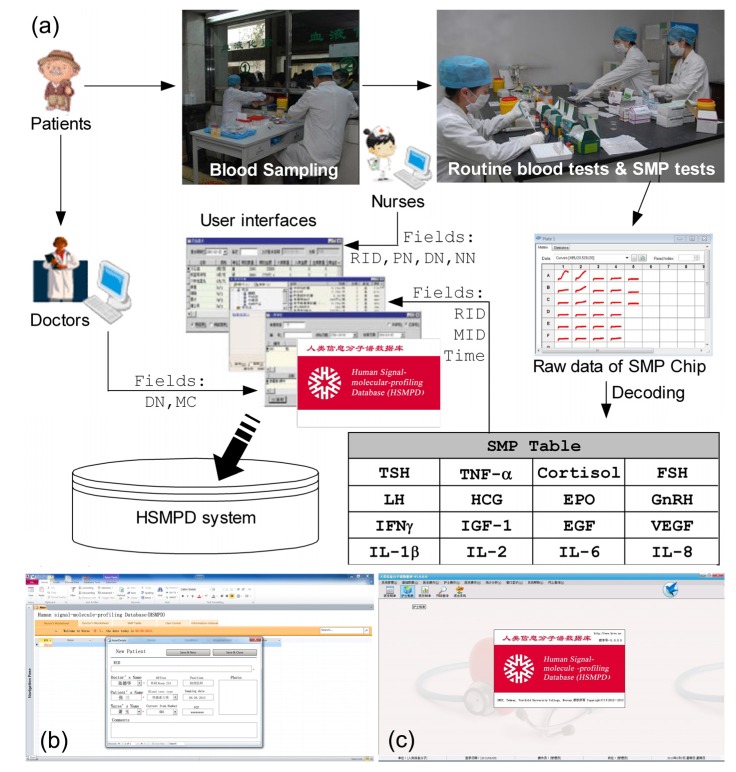
Typical process of data acquisition in HSMPD system. (**a**) A flow chart above is to demonstrate the data acquisition process for HSMPD. Only 16 types of signal molecule targets were measure on the SMP chip prototype in this study. (**b**) The first version of HSMPD system was developed by Microsoft Access^®^ 2010. (**c**) The second version of HSMPD system was made in Microsoft^®^ SQL Server^®^ 2005.

This simulation tests were supported by a hospital, which procedures were shown in Figure 5. In total 200 SMP chip units were provided as new tools for ordinary blood testing to clinical laboratories in the cooperating hospital. Given that the testing samples for SMP chips were only diluted serums, a small volume of blood samples was enough for a single SMP test, which means that the remaining sample left from an ordinary blood test is usually sufficient for an additional SMP test. When patients are advised by doctors to examine the blood, the patients will go to the blood-collection room for blood sampling. Our researchers were permitted to wait in the room and give the patients suggestions to permit the additional SMP tests accompanied by their original blood tests. The data acquisition was performed in a passive way, *i.e.*, the participators were randomly involved in.

We orally interviewed the patients, doctors and nurses regarding their attitude towards the SMP test. The investigators for survey studies were divided into two groups: one group interviewed the people who will get their blood tested in the blood-collection room of hospitals, whereas the other group randomly interviewed the pedestrians in city streets far away from hospitals (Ziyang City, China). The subjects for this investigation were randomly selected and unfiltered. The time period of each group was consistent and was evenly distributed in two weeks. We made further inquiries 189 amicable interviewees who were willing to take the SMP tests about their background, as well as the privacy protection in SMP data. The patients were fully informed before the voluntary agreements for SMP tests were signed. The researchers obtained the respective remaining samples from nurses after the ordinary blood tests were performed. No extra sampling of blood was performed in this study.

The SMP tests were handled by our researchers, and the decoded results were imported into the demo database system within the intranet of the hospital (Figure 5b,c). The involved patients, doctors and nurses participated in scientific investigations related to the tests and the demo system. Their comments about the demo HSMPD system were collected. The name and symptoms of the patients were inputted into the demo system. The doctors provided the patients their SMP information when necessary. After the study, the demo database was deleted from the local network; no private information of the patients was divulged outward as we promised.

### 2.5. Information Retrieval Tests in the Demo Database

We simulated a TBI researcher assigned to retrieve information from the HSMPD for optimization of the HSMPD system. Both fields of SMP values and medical comments were employed for the retrieval tests, which results were simply grouped into initial subsets. Moreover, we read the entire description in MC to manually find out the missing records of the above subsets. The causes of retrieval failures were analyzed.

## 3. Results and Discussion

### 3.1. Survey Studies in Data Acquisition

**Figure 6:**
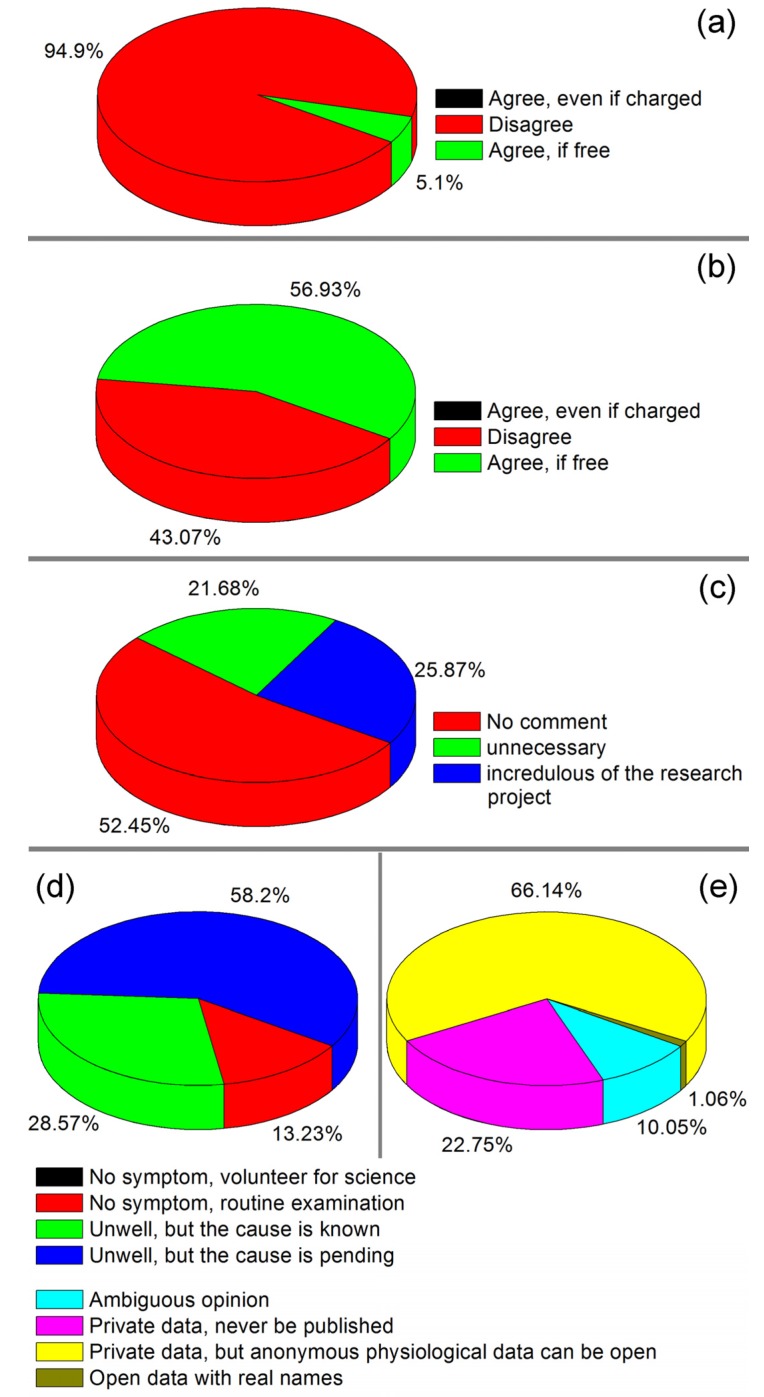
Survey study in the data acquisition process of HSMPD [34]. (**a**) The survey result on the attitudes towards HSMPD among the people far away from hospitals. (**b**) The different survey result on the attitude of the public in the blood-collection room. (**c**) The distribution of reasons given by 143 interviewees who refused to participate in the HSMPD project. (**d**) The reasons and purposes of the 189 participants in the HSMPD project. (**e**) The points of view of the participants regarding the privacy issues of the HSMPD.

The survey results about the patients’ attitude towards HSMPD were presented in Figure 6. Figure 6a shows that over 90% of the general informants far away from hospitals did not want to participate or refused to participate in the SMP tests, even if the tests were free of charge. The result in the blood-taking room was significantly different. Figure 6b shows that more than half of the patients were willing to accept the free SMP tests without additional blood sampling. However, no one was willing to participate if the SMP tests were not free. This survey may not have been rigorous enough, but the conclusion is logical: it is impossible to ask the patients to pay for the SMP tests. Figure 6c shows the reasons given by 143 patients who refused to participate in the project. More than half of the patients were distrustful of investigators and were unwilling to be interviewed. The reasons for the rejection can be divided into two: first, the patients considered the SMP tests useless for them; second, the patients were distrustful of this scientific project.

Figure 6d shows the reasons and purposes of taking the blood tests. Among the interviewees, 86.77% had uncomfortable symptoms and needed medical examination under the doctor’s advice. Moreover, 58.2% of the informants did not know the cause of their disease. The other 13.23% claimed to be undergoing routine physical examinations and meanwhile took the free SMP tests. We did not meet any person who claimed to take the SMP tests solely for the purpose of scientific research. The result in Figure 6d indicated that the people who participate in scientific projects subconsciously hope to obtain additional information from the SMP tests. It can be concluded that the people likely to accept the SMP tests only when they spontaneously want to take blood tests. Figure 6e shows the points of view of the users who participated in the SMP tests regarding the privacy issues of the HSMPD. The main opinion of the users was that research data should not involve private information, but physiological information should be available for disclosure. Moreover, 22.75% of the informants refused to open their own data. Doctors and nurses also gave some comments about the demo HSMPD system and user interfaces, which are listed in Table 1.

**Table 1:** Comments on the demo HSMPD system from doctors and nurses (six doctors and four nurses participated).

No.	Comments	Possible Scenario	Reviewers
1	Valuable medical comments should not be free.	Doctors will be rewarded for their contributions in the future.	Doctors
2	A SMP record includes too many items and numbers, so some abnormal data might be overlooked.	The SMP data will be preliminarily analysed before they are delivered to doctors. The abnormal items will be highlighted.	Doctors
3	The automation and intelligence of the input interface should be increased.	The drop-down boxes will be introduced in the user interface for easy input.	Doctors, Nurses
4	The names of patients or doctors may be not unique, which often results in problems.	The ID number of patients and doctors may be employed to replace their names.	Nurses
5	The ID number of the chip may be too long and not easy to input correctly.	The bar code reader will be used to input the Chip ID.	Nurses
6	The manual operation of the SMP chip is too complex and should be improved.	The automatic device for the SMP chip will be easy to use.	Nurses

Before this survey, the roadmap of HSMPD had two different versions. In version A, it was presumed that the patients would pay for the costs of their SMP tests, as long as SMP chips were cheap enough. Consequently, it is not necessary to commercialize any medical data, and many ethical problems can be avoided. However, plan B disagreed with the previous assumption. In version B of the roadmap, commercialization of the medical database is essential to balance income and expenses during the construction of HSMPD. The survey results clearly demonstrated that only version B is practical. The paid services of medical data in HSMPD have to be included in the roadmap of HSMPD, although more ethical issues will be caused by this action.

Since the mode of data collection in the HSMPD project is passive, it does not allow the selection of test objects. The passive mode is unable to meet the needs of medical research, particularly experimental research in pharmacy. Generally, medical experiments include making assumptions, designing animal experiments between the control group and the experimental group, and conducting clinical trials to verify the hypotheses. For the short term, the HSMPD cannot fulfill the need of designing experiments. However, the HSMPD may help medical experimental research in the long run. When sufficient data accumulated in HSMPD, the medical assumptions will be easier to check by the rapid statistical analysis on the clinical cases in HSMPD.

### 3.2. Information Retrieval Tests

The SMP value for each patient was a multivariate set of real numbers, thus making this value suitable for storage in a multi-field table. The SMP measurement was imported into the database artificially. In the future, these data will be directly uploaded into the database by computers, thus simplifying the storage and retrieval of SMP values.

MC filed is a character string inputted by doctors, which is easy to store but difficult to retrieve correctly. We also met some challenges when retrieving the MC Field. Searching for keywords in MC is easy, and the retrieval results can be simply grouped into initial subsets. However, we found some missing records upon reading the entire description in MC. Some doctors input many synonyms and some abbreviations in the MC Field, thereby causing possible retrieval failures. The majority of doctors in this study choose Chinese as their working language, but few doctors partly used English or Latin abbreviations in their comments. In addition, comments with positive or negative tones should roughly divide the previous subset into three subclasses: positive, suspected, or negative. The method to identify the positive or negative tone in the MC is another challenge in the creation of the HSMPD. Much energy and time was spent on segregating the comments here. The HSMPD might employ an intelligent user-interface of other databases to bypass this challenge.

### 3.3. Validation of SMP Chips

**Figure 7:**
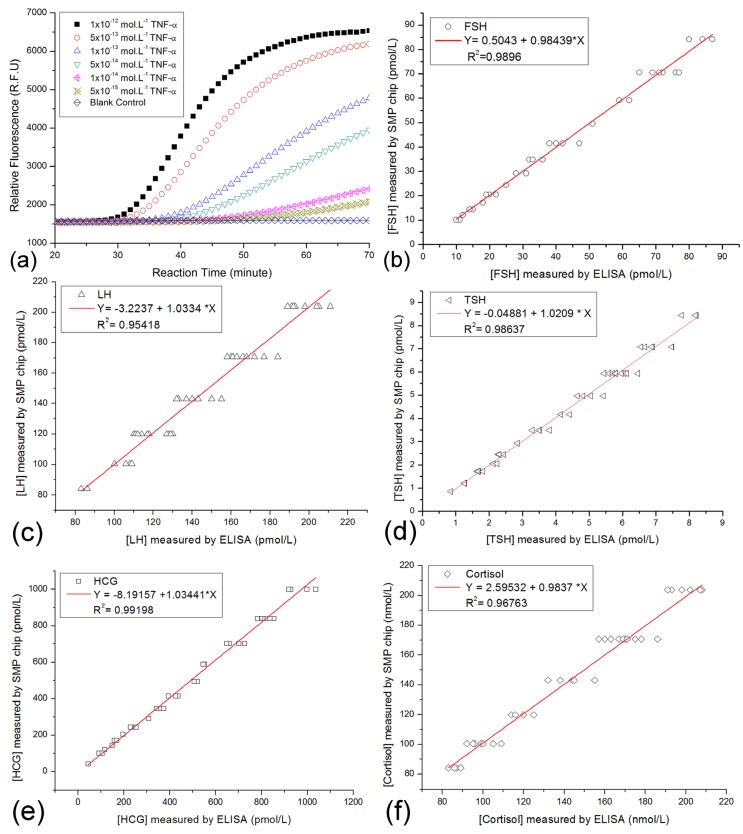
Comparison plots for corresponding results of both the on-chip SMP testing and traditional ELISA method. (**a**) Plot A showed the typical curves of on-chip immuno-NASBA assays using artificial TNF-α solutions as the standard samples. The decoding of the raw date in the curves was described before [33]. Multiple signal molecule targets in patient samples were measured on the SMP chips, while some patient samples were also quantified by the other ELISA kits. (**b**) The comparison plots of the test results of FSH. (**c**) The comparison plots of the test results of LH. (**d**) The comparison plots of the test results of TSH. (**e**) The comparison plots of the test results of HCG. (**f**) The comparison plots of the test results of cortisol.

Normalized measurement of on-chip immuno-NASBA assay could provide comparative data as the traditional ELISA methods. In this study, only 189 volunteers participated in the SMP tests. Then, 189 SMP records were collected for the database. As mentioned above, the clinical samples were measured by ordinary blood tests before the testing on SMP chips. Some ordinary blood tests included some quantification ELISA tests for hormones. The comparison plots of five common targets from both SMP chip testing and ELISA methods were shown in Figure 7.

FSH, LH, TSH, HCG and cortisol in about 40 patient specimens were measured by the two methods. From the comparison plots in Figure 7, we found the differences of results from two methods were small. It implied that the correctness of the SMP chip was verified, but the precision of SMP chips needs further improvement. Given that the storage capacity of the demo HSMPD was too small, few records were classified into their corresponding disease category. Thus, performing data mining was not applicable in this case.

### 3.4. Discussion on the Data Structure of HSMPD

The HSMPD data structure was optimized according to the comments about private information of patients. User information was divided into two sections. The privacy section cannot be accessed without the mediation of a trusted intermediary, whereas the public part is allowed to contain SMP values and medical comments from doctors. The identity information of patients is replaced by a string of numbers, PCP, which could make each clinical record anonymous to scientists. In addition, the PCP is also a pseudonym of each patient. PCP can help the TBI researchers to distinguish individual records in HSMPD and retain a clue for in-depth studies when it is necessary. To some important patients or doctors, TBI researchers could indirectly contact them through the trusted intermediary according to the PCPs. If the patients or doctors are willing to participate in the in-depth study, the researchers can access detailed clinical data. The role of PCP may then be acceptable for both parties.

The input of MC in HSMPD seemed to be a challenge. From the interviews with doctors, the feedback shows their disfavour of electronic recording for the HSMPD. They prefer to use the traditional way of inputting their comments in the database system installed in the hospital. Moreover, doctors were unwilling to spend any time on filling unnecessary forms. To avoid the typing of medical comments, as some doctors suggested, the HSMPD interfaces could be merged into the local hospital information system (HIS), hence the medical records of the patients could be exported into the HSMPD automatically. Many HIS software applications have been commercialized. One of them was used inside the cooperating hospital, which has a well-designed database system to organize the daily work in the hospital. Most fields in the HSMPD, such as PN, DN, NN, MC and Time, have been built into the HIS. The HSMPD software of the next version might become an add-in program of current HIS system, which interfaces should strictly fit the current work habits of doctors and nurses.

### 3.5. Technical Issues in the HSMPD

Actually, HSMPD was proposed on the basis of fundamental physiological theories. In the past decades, biologists always tried to find a simplex biomarker to represent the status of a specific disease, but such a one-variable function maybe never exist in the case of many complex diseases. However, the biologists have no alternative because few qualified serological database is available for data mining. Being a perspective database, HSMPD is an open data warehouse of serological data with medical comments. It is designed and standardized for TBI research, rather than the common healthcare processes. The outbreak of TBI applications in the serological diagnosis might appear after the completion of HSMPD. 

However, reducing the cost of data acquisition is crucial. The SMP chip can be considered a low-throughput protein microarray with a 3D structure, which might enhance the efficiency of the immunoassay. Given that low-cost materials and fabrication methods were employed, the cost of the SMP chip did not increase significantly compared with that of traditional microarrays. Shrinking the chips might save expensive antibodies, but the cost of detection modules will increase consequently. The compatibility of SMP chips provides multiple choices of detection modules. The current detection module was a microplate reader, whereas a more professional SMP-chip reading platform is still being developed potentially on the basis of our previous research achievements [35,36,37]. Unless a technical breakthrough occurs in the development of HSMPD-oriented tools, the construction of HSMPD has to rely on the support from multidisciplinary cooperation. Any technological progress might accelerate the construction process.

### 3.6. Nontechnical Challenges in HSMPD Construction

The establishment of a public HSMPD database will greatly facilitate TBI research on the association between diseases and serological patterns of patients, because scientists could study millions or billions of standard clinical SMP records in the HSMPD through biostatistics programs on a national network platform, instead of investigating hundreds of patients individually. From the viewpoint of scientists, the creation of HSMPD will significantly assist in medical research, particularly in disease diagnosis and chronic disease management.

Although HSMPD has built in protection design for the privacy of participants, tremendous ethical and social controversy may be generated between scientists and participants of the HSMPD. Anonymous records have to be employed the infrastructure of the HSMPD, which means that the database will not release the fields of non-essential personal information, such as name, home address and birthday. Only physiological and medical information will be shared in the public database. Traditional consent norms and policies require participants to be fully informed of the nature and risks of the research [38]. However, considering the large number of participants and the long-term nature of the HSMPD, obtaining specific consent from millions of participants for each study is impossible [39]. A logical consequence is to seek alternative approaches such as having a trusted intermediary that consents on behalf of the participants, e.g., government departments. The hidden fields involving the private information of patients may be used to disclose the real identity of patients; therefore, those fields should be legally protected. Abuse of the data in the HSMPD could be prevented under legal supervision.

Moreover, the construction of the HSMPD depends on a huge investment, even if it is far less than the cost of HGP. We suggested a construction mode with minimum investment. This project needs to have relatively small investments at the early stage. However, given that the HSMPD relies on the information resource of the database, the HSMPD needs to acquire income to maintain its growth. This stage has entered a cycle of positive feedback. The larger the capability of HSMPD is, the more abundant the funding from the IT service becomes. When the HSMPD matures, commercialized HIT services will continue to bring a virtuous cycle for the entire system, whereas TBI researchers might obtain more resources for further study.

## 4. Conclusions

The construction of high-quality clinical database might be the main bottleneck at present for the development of TBI. The HSMPD, an ideal clinical database, is designed for TBI research and supported by its standardized tool for data acquisition. The clinical records in HSMPD could be a promising resource to significantly influence the TBI research. However, nontechnical challenges mainly hinder the construction of HSMPD, including the high cost of data acquisition and some ethical issues. By comparison, the technical aspect of HSMPD project is not very challenging. A feasible solution was proposed to build the HSMPD with minimum investment, which relies on the cooperation of IT, instrumentation engineering, health care systems and the TBI community. The SMP chip and the demo HSMPD system were developed and preliminarily evaluated in the hospital. The positive results in this study demonstrated a rational and acceptable mode for the construction of HSMPD, which will play a role as the prospective bridge between traditional health care systems and the TBI community.

## List of Abbreviations

DN: Doctor’s Name
EGF: epidermal growth factor
ELISA: enzyme-linked immunosorbent assay
EPO: erythropoietin
FSH: follicle-stimulating hormone
GnRH: gonadotropin-releasing hormone
IFNγ: interferon-gamma
IGF-1: insulin-like growth factor 1
HCG: human chorionic gonadotropin
HER: electronic health record
HGD: Human Genome Database
HGP: Human Genome Project
HIS: Hospital Information System
HIT: Health information technology
HSMPD: human signal-molecular-profiling database
IL: interleukin
Immuno-NASBA: immuno-nucleic acid sequence-based amplification
LH: luteinizing hormone
MC: medical comments
MID: Machine ID
NN: Nurse’s Name
PCP: Personal Code of Patient
PN: Patient’s Name
RID: Record ID
SMP: signal molecular profiling
TBI: translational bioinformatics
TNF-α: tumor necrosis factor-alpha
TSH: thyroid-stimulating hormone
VEGF: vascular endothelial growth factor

## References

[1] Sittig D.F., Wright A., Simonaitis L., Carpenter J.D., Allen G.O., Doebbeling B.N., Sirajuddin A.M., Ash J.S., Middleton B. (2010). The state of the art in clinical knowledge management: An inventory of tools and techniques. Int. J. Med. Inform..

[2] Biobank Norway. http://www.ntnu.edu/nb/web/biobanknorway.

[3] Cancer Registry of Norway. http://www.kreftregisteret.no/en/research/Janus-Serum-Bank/.

[4] Thomas R., Phuong J., McHale C.M., Zhang L. (2012). Using bioinformatic approaches to identify pathways targeted by human leukemogens. Int. J. Environ. Res. Public Health.

[5] Colantonio S., Esposito M., Martinelli M., De Pietro G., Salvetti O. (2012). A knowledge editing service for multisource data management in remote health monitoring. IEEE Trans. Inf. Technol. Biomed..

[6] Lista S., Faltraco F., Hampel H. (2013). Biological and methodical challenges of blood-based proteomics in the field of neurological research. Prog. Neurobiol..

[7] Ekeland A.G., Bowes A., Flottorp S. (2012). Methodologies for assessing telemedicine: A systematic review of reviews. Int. J. Med. Inform..

[8] Cheng P.-H., Lin B.-S., Yu C., Hu S.-H., Chen S.-J. (2013). A seamless ubiquitous telehealthcare tunnel. Int. J. Environ. Res. Public Health.

[9] Brown J.A. (2010). Using a relational database to index infectious disease information. Int. J. Environ. Res. Public Health.

[10] Thiele I., Swainston N., Fleming R.M., Hoppe A., Sahoo S., Aurich M.K., Haraldsdottir H., Mo M.L., Rolfsson O., Stobbe M.D. (2013). A community-driven global reconstruction of human metabolism. Nat. Biotechnol..

[11] Sorani M.D., Ortmann W.A., Bierwagen E.P., Behrens T.W. (2010). Clinical and biological data integration for biomarker discovery. Drug Discov. Today.

[12] Knox C., Law V., Jewison T., Liu P., Ly S., Frolkis A., Pon A., Banco K., Mak C., Neveu V. (2011). DrugBank 3.0: A comprehensive resource for ‘Omics’ research on drugs. Nucl. Acids Res..

[13] Wishart D.S., Jewison T., Guo A.C., Wilson M., Knox C., Liu Y., Djoumbou Y., Mandal R., Aziat F., Dong E. (2013). HMBD 3.0—The human metabolome database in 2013. Nucl. Acids Res..

[14] Bodmer W., Maloy S., Hughes K. (2013). Human Genome Projec. Brenner’s Encyclopedia of Genetics, Volumes 1–4.

[15] ClinicalTrails.gov. http://www.clinicaltrials.gov/ct2/home.

[16] Pickett B.E., Greer D.S., Zhang Y., Stewart L., Zhou L., Sun G., Gu Z., Kumar S., Zaremba S., Larsen C.N. (2012). Virus pathogen database and analysis resource (ViPR): A comprehensive bioinformatics database and analysis resource for the coronavirus research community. Viruses.

[17] Torri F., Dinov I.D., Zamanyan A., Hobel S., Genco A., Petrosyan P., Clark A.P., Liu Z., Eggert P., Pierce J. (2012). Next generation sequence analysis and computational genomics using graphical pipeline workflows. Genes.

[18] Geissbuhler A., Safran C., Buchan I., Bellazzi R., Labkoff S., Eilenberg K., Leese A., Richardson C., Mantas J., Murray P. (2013). Trustworthy reuse of health data: A transnational perspective. Int. J. Med. Inform..

[19] Wilson S.J., Wong D., Clifton D., Fleming S., Way R., Pullinger R., Tarassenko L. (2013). Track and trigger in an emergency department: An observational evaluation study. Emerg. Med. J..

[20] Hayrinen K., Saranto K., Nykanen P. (2008). Definition, structure, content, use and impacts of electronic health records: A review of the research literature. Int. J. Med. Infor..

[21] Caccia D., Dugo M., Callari M., Bongarzone I. (2013). Bioinformatics tools for secretome analysis. Biochim. Biophys. Acta.

[22] Magliano D.J., Rogers S.L., Abramson M.J., Tonkin A.M. (2006). Hormone therapy and cardiovascular disease: A systematic review and meta-analysis. BJOG Int. J. Obstet. Gynaecol..

[23] Kim W.G., Cheng S.-Y. (2013). Thyroid hormone receptors and cancer. Biochim. Biophys. Acta.

[24] Mendonsa G., Dobrowolska J., Lin A., Vijairania P., Jong Y.-J.I., Baenziger N.L. (2009). Molecular profiling reveals diversity of stress signal transduction cascades in highly penetrant Alzheimer’s disease human skin fibroblasts. PLoS One.

[25] Carey M.A., Card J.W., Voltz J.W., Germolec D.R., Korach K.S., Zeldin D.C. (2007). The impact of sex and sex hormones on lung physiology and disease: Lessons from animal studies. Am. J. Physiol..

[26] Dong T., Zhao X., Yang Z. Concept and Approach of Human Signal-Molecular-Profiling Database: A Pilot Study on Depression Using Lab-On-Chips. Proceedings of the 2013 35th Annual International Conference of the IEEE Engineering in Medicine and Biology Society (EMBC).

[27] Zhao X., Dong T., Yang Z., Pires N., Hoivik N. (2012). Compatible immuno-NASBA LOC device for quantitative detection of waterborne pathogens: Design and validation. Lab Chip.

[28] Pires N.M.M., Dong T., Yang Z., Hoivik N., Zhao X. (2011). A mediator embedded micro-immunosensing unit for electrochemical detection on viruses within physiological saline media. J. Micromech. Microeng..

[29] Zhang L., Dong T. (2013). A Si/SiGe quantum well based biosensor for direct analysis of exothermic biochemical reaction. J. Micromech. Microeng..

[30] Dong T., Yang Z., Su Q., Nhut M.T., Egeland E.B., Karlsen F., Zhang Y., Kapiris M.J., Jakobsen H. (2011). Integratable non-clogging microconcentrator based on counter-flow principle for continuous enrichment of CaSki cells sample. Microfluid Nanofluid.

[31] Zhao X., Dong T. (2013). Design and fabrication of low-cost 1536-chamber microfluidic microarrays for mood-disorders-related serological studies. Sensors.

[32] Chen C.-Y., Chang C.-L., Chang C.-W., Lai S.-C., Chien T.-F., Huang H.-Y., Chiou J.-C., Luo C.-H. (2013). A low-power bio-potential acquisition system with flexible PDMS dry electrodes for portable ubiquitous healthcare applications. Sensors.

[33] Zhao X., Dong T. (2012). Multifunctional sample preparation kit and on-chip quantitative nucleic acid sequence-based amplification tests for microbial detection. Anal. Chem..

[34] Zhao X., Dong T.  Feasibility Study on Service-Based Data Acquisition for Human Signal Molecule Profiling Database. Proceedings of the 13th IEEE International Conference on BioInformatics and BioEngineering.

[35] Pires N.M.M., Dong T., Hanke U., Hoivik N. (2013). Integrated optical microfluidic biosensor using a polycarbazole photodetector for point-of-care detection of hormonal compounds. J. Biomed. Opt..

[36] Zhao X., Dong T. (2013). A microfluidic device for continuous sensing of systemic acute toxicants in drinking water. Int. J. Environ. Res. Public Health.

[37] Pires N.M.M., Dong T. (2013). Microfluidic biosensor array with integrated poly(2,7-carbazole)/fullerene-based photodiodes for rapid multiplexed detection of pathogens. Sensors.

[38] Master Z., Nelson E., Murdoch B., Caulfield T. (2012). Biobanks, consent and claims of consensus. Nat. Methods.

[39] Petrini C. (2010). Theoretical models and operational frameworks in public health ethics. Int. J. Environ. Res. Public Health.

